# How stupid has science been?

**DOI:** 10.1038/s44319-025-00562-x

**Published:** 2025-08-26

**Authors:** Arthur Caplan

**Affiliations:** Division of Medical Ethics, NYU Grossman School of Medicine, New York, NY USA

**Keywords:** Economics, Law & Politics, History & Philosophy of Science, Science Policy & Publishing

## Abstract

The public’s reluctance to come to the defence of scientists under attack by the US government is in good part to blame on scientists themselves.

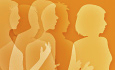

Watching mainstream science in America under attack by the US federal and many state governments, most scientists and health care providers are wondering how this could be happening. The USA is a nation with an amazing history of research and technological innovation, and its impressive economic growth is linked directly to advances in science and technology. Historically, the nation has made heroes of its scientists; Franklin, Carver, Bell, Deere, Edison, Ford, the Wright Brothers, Einstein, Feynman and many more. So how can US President Trump, Secretary of Health Robert F Kennedy Jr or Director of the Centers for Medicare and Medicaid Mehmet Oz and their enthusiastic followers be succeeding in defunding research and installing ideological oversight and censorship that is crushing science, technology and engineering and will for many years to come?

Part of the answer is that US science itself is to blame. It has disparaged its public communication as unnecessary and looked down on those few who tried to educate broader audiences about the wonders, benefits, methods and advancements of science. The belittling of the astronomer and planetary scientist Carl Sagan is a prime example. Consider this interview with Robert M Sapolsky, then a professor of biology, neurology, and neurosurgery at Stanford University and a popularizer of work in neuroscience. He was asked: “Do you feel a tension as an academic doing this kind of [popular] writing?” Sapolsky replied, “Tremendous amounts of tension. It does take up a lot of time. On the other hand, most of my science peers have a lot of their time taken up by being on this committee or chairing this program or being editor of this or that journal, so I would much rather be doing this with my ‘non-direct science time.’ But there’s a funny science culture about this stuff… the sort of snotty term for it is that you get *Saganized*. People decide you can’t possibly be doing serious science anymore if you are also spending time trying to communicate about it.”

Or consider the theoretical physicist Sean Carroll commenting on the risks of popularizing on careers in science: “Do not be too well known outside the field. I hate to say this, but the evidence is there: if you have too high of a public profile, people look at you suspiciously.” Sapolsky and Carroll capture the contempt and intolerance much of mainstream science has had for those speaking off-campus to non-science audiences. The resulting failure to communicate about science to the public is a major factor in explaining why so few have rallied to science’s defense today against government policies promoting ignorance, illiteracy and quackery.

In 1991, Sagan was nominated for membership in the US National Academy of Sciences for his work in astronomy. He was not elected despite the efforts of some strong backers, including the Nobel laureate Stanley Miller, who advocated passionately for Sagan’s admission (Martinez-Conde, 2016). Sagan secured less than 50% of the “yes” votes, far from the two-thirds required for admission to the Academy. Sagan’s biographers have argued that the Academy’s rejection of Sagan, and Harvard’s prior denial of tenure to him, were the direct consequence of the phenomenon now known in his name as ‘Saganization’ or the “Sagan Effect”: the perception that highly visible scientists who successfully communicate with the public must do so due to their research being inferior or lacking the commitment of time and attention that top-tier science demands. Popularizers engage in activities that are indicative of research failure, dangerously oversimplify, are self-indulgent and produce no tangible benefits.

The disparagement Sagan suffered was indefensible. Analyses of Sagan’s academic output find that his contributions compared favorably to those of other Academy members. “Throughout his career, which began in 1957 and ended in 1996, […] Sagan averaged a scientific, peer-reviewed paper per month” (Shermer, 1999). Still, Sagan’s peers in his scientific specialty and in the sciences generally frequently disparaged his efforts at popularizing science, especially when he did so through television appearances. His own scientific work was discounted, not due to its quality or quantity, but by dint of association with an author eager and committed to popularizing science and an elitist belief that an unsophisticated public was not worth the time of truly top-flight minds.

Sagan was not the only scientist to see their work as a popularizer denigrated and their competency challenged. One of the first thing critics of evolutionary biologist Stephen Jay Gould’s views brought up was his activity as a popularizer. Again and again, those who disagreed with him asserted that Gould was just a science popularizer not a real scientist. He was a lightweight writing essays and books that could not have rested on his scientific acumen and brilliance because they were clear, engaging and popular. This is sheer nonsense, but it is important to note that in heated battles over the validity of evolutionary psychology, sociobiology and IQ testing the charge of popularizer was a handy weapon to use to dismiss him.

The price for years and years of unwarranted, misguided snobbery is now being paid. Populists and right-wing thinkers have been losing faith in science for years. “Since the 1980s, trust of science among conservatives in America has been plummeting” (Gligorić et al, 2025). It is no surprise then that, outside of those directly impacted, the public response to this systematic, outrageous and devastating attack has been sadly, at best, muted. The American people, now egged on by anti-intellectualist, anti-expertise and anti-science politicians and traumatized by the horrors of the Covid pandemic, blaming mainstream science sometimes for causing it and frequently for failing to promptly thwart it despite demanding burdensome restrictions.

Scientists seem stunned by the ferocity of the attack on their expertise by an army of unqualified voices and the lack of a loud, spirited defense of their work. However, if members of the public do not know much about science, haven’t met a scientist, never have heard a scientist, see far more frauds and charlatans speaking and tweeting forcefully with indifference to the appropriate science or contradicting it, if few within science are trained to communicate about their work and those who do are not only not rewarded but disparaged, then it is not as hard to see why the war on science has been prosecuted with little resistance.

## Supplementary information

Peer Review File
