# Supplementary material for: How stupid has science been?
Source: EMBO Rep. 2025 Aug 26;26(18):4395–6. doi: 10.1038/s44319-025-00562-x (PMC12457644; doi:10.1038/s44319-025-00562-x)
Supplement: Supplementary file 1 — Peer Review File [file 44319_2025_562_MOESM1_ESM.pdf]

EMBO reports

How stupid has science been?

Arthur Caplan

Corresponding author(s): Arthur Caplan (Arthur.Caplan@nyumc.org)

Review Timeline:

Submission Date:	14th Aug 25
Accepted:	15th Aug 25

Editor: Holger Breithaupt

Transaction Report: This article type is not peer reviewed.
